# Maternal Touch Moderates Sex Differences in Juvenile Social Play Behavior

**DOI:** 10.1371/journal.pone.0057396

**Published:** 2013-02-27

**Authors:** Michelle N. Edelmann, Catherine H. Demers, Anthony P. Auger

**Affiliations:** 1 Neuroscience Training Program, University of Wisconsin-Madison, Madison, Wisconsin, United States of America; 2 Psychology Department, University of Wisconsin-Madison, Madison, Wisconsin, United States of America; University of Granada, Spain

## Abstract

Additional somatosensory contact of preterm human infants improves a variety of developmental assessment scores, but less is known about its lasting consequences. In rodents, maternal contact may influence the programming of juvenile social play behavior. Therefore, we used a paradigm where we can control the levels of somatosensory contact associated with maternal care. We find that additional somatosensory contact of offspring can have lasting consequences on juvenile social play behavior in a sex-dependent manner. Specifically, additional somatosensory stimuli reduced male social play behavior, but did not change female play behavior. We then examined if this additional infant contact altered some neurobiological substrates associated with play within the juvenile amygdala. Control males had lower levels of 5HT2a receptor mRNA levels contrasted to females; however, similar to its sex-dependent effect on juvenile social play, males that received additional somatosensory contact had higher serotonin 5HT2a receptor mRNA levels than control males. No difference was found in females. As serotonin signaling typically opposes juvenile play behavior, these data suggest that maternal touch can program lasting differences in juvenile social play and 5HT2a receptors mRNA levels within the juvenile amygdala.

## Introduction

While there is a large volume of literature examining the lasting consequences of early maternal separation and stress on newborn offspring, there are some studies suggesting the importance of somatosensory contact during infant development [1]. For example, institutionalized infants show improvement on developmental assessment scores following 20 minutes of additional tactile stimulation per day for 10 weeks [2]. Other studies report dramatic developmental improvement of premature infants receiving additional tactile stimulation while in neonatal intensive care units [3]. These studies illustrate the importance of additional infant contact during development, but less is known about the lasting consequences of infant touch. Indeed, some studies suggest that maternal contact may be particularly important in programming juvenile behavior, such as social play.

Rodent studies that have examined whether maternal care shapes social play have primarily focused on differences in the amount of anogenital licking and grooming (LG) given by the mother. That is, mother rats preferentially lick and groom male offspring more than female offspring [4], and this tactile stimuli seems to be important for programming differences in juvenile social play behavior. Past studies have given dams peripheral zinc sulfate or dietary saline to specifically reduce maternal anogenital grooming [5]. It was found that male rats from dams that gave more LG during the neonatal period engaged in lower juvenile social play. More recently, research examining natural variations in maternal care found that male offspring from dams exhibiting increased levels of pup licking/grooming and arched-back nursing (high LG-ABN mothers) engaged in less social play than males from low LG-ABN mothers [6]. Although these studies suggest that variations in maternal care influence play behavior, it is important to note that the dams in these studies also displayed other differences in maternal care, like time spent in the nest or the time spent arched-back nursing. As such, it is still unclear whether the somatosensory stimulation associated with maternal grooming is specifically responsible for the differences seen in juvenile social play behavior.

Juvenile social play behavior in rats, which is thought to be one of the earliest non-mother directed social behaviors, involves a complex set of behaviors that seem to be modulated by several brain regions and neurobiological systems [7], [8]. The brain regions that influence juvenile play behavior have mainly been elucidated by lesion studies. For example, damaging the cortex, mediobasal hypothalamus, nucleus accumbens, or amygdala will result in decreased social play [8]. The importance of the amygdala has been further elucidated by studies that examine region specificity. During the juvenile period, males engage in higher levels of social play behavior than females [9], [10], and testosterone treatment directly into the developing amygdala is sufficient to fully masculinize social play behavior in females [11]. This suggests that the amygdala plays an important role in sexually differentiating juvenile social play behavior.

While organization of sex differences in juvenile social play behavior is influenced by the endogenous steroid hormones [11]–[16], other research has indicated that neurotransmitters are also important in controlling juvenile social play behavior, such as dopamine and serotonin. Indeed, pharmacologically altering dopamine signaling can enhance juvenile social play behavior [16]–[19]; whereas, serotonin (5-hydroxytryptamine, 5-HT), which plays an important role in the regulation of aggression [20], [21], appears to have an inverse relationship with social play behavior [22]. Although several neurobiological substrates can regulate social play, it is unclear how maternal care impacts these systems to alter juvenile social play.

The following study examines whether only manipulating the amount of somatosensory stimulation associated with maternal grooming is sufficient to alter juvenile social play and some neurobiological substrates associated with juvenile social play within the juvenile amygdala.

## Methods

### Ethics statement

This research was approved by the University of Wisconsin Animal Care and Use Committee.

### General Care of Animals

Adult Sprague-Dawley rats (Charles River Laboratories, Inc., Wilmington, MA) were kept in our animal facility on a 12 hour light∶ 12 hour dark cycle. Food and water were available *ad libitum*. Adult female rats were mated and allowed to deliver normally. Cages were checked regularly to determine the day of birth. Day of birth is considered postnatal day 0 (P0). Litters were culled to ten pups each. On P0, one or two feet from each pup were tattooed with a small amount of India ink for identification purposes. Each treatment group was represented by at least two pups in every litter.

### Simulated maternal grooming (SMG) paradigm

Neonatal rats received simulated maternal grooming (SMG) or control handling three times per day, one in the light cycle and two in the dark cycle, from P0 until P10. During treatment, all neonatal rats were removed from the dam immediately prior to treatment, kept under a warming lamp and on a warm heating pad while away from the dam, and immediately returned to the dam following treatment. SMG consisted of 3 rounds of 10 strokes to the anogenital region with a soft nylon bristled brush. Control pups were handled in the same manner but did not receive tactile stimulation from the paintbrush. Time away from dam was about 15 minutes or less. In order to control for differences in maternal care, pups alternated between dams after each SMG session. Each litter was composed of both sexes, as well as treatment and control groups. This allows for each dam to care for a mixed litter of sexes, SMG-treated, and control-handled pups. The SMG paradigm is identical for both experiments.

### Experiment 1: Does simulated maternal grooming influence juvenile social play behavior?

#### Juvenile Social Play Behavior (JSP)

The social play behavior paradigm was similar to those previously published [11], [16], [23]. After P10, pups were left undisturbed until weaned from the dam at P21. During weaning, rats were housed in groups of six or seven containing animals from all treatment groups. Animals were maintained in these groups throughout the experiment. Animal tails were coded with a Sharpie marker to identify individuals. Rough-and-tumble social play behavior was scored on PN26–29, using a paradigm adapted from [16]. Animals were video recorded for two 4 minute trials per day over 4 days, with one trial occurring 2 hours after lights-off and one trial occurring 4 hours after lights-off, for a total observation time of 32 minutes per animal. All animals were videotaped in their home cages. The tapes were scored by an observer blind to the treatment groups. The total amount of play behavior was calculated by totaling the number of times each animal engaged in biting, pinning, or pouncing over the entire observation time. Previous studies were used to determine the scoring criteria for specific types of play behavior published [11], [16], [23]. Specifically, biting was scored when one rat grabbed another rat's skin with its mouth. Pouncing was scored when one rat lunged and put its forepaws on the dorsal side of another rat. Pinning was scored when one rat stood over another with its forepaws on the ventral side of the opposing rat. In experiment 1, there were 8 control females, 9 SMG females, 9 control males, and 8 SMG males from 4 litters with each treatment group represented by at least two pups in every litter.

### Experiment 2: Does SMG alter neurobiological substrates associated with JSP?

#### Tissue Collection

On postnatal day 25 (P25), brains were collected, snap frozen, and stored at −80°C. Each brain was then sectioned at 250 µm using a cryostat (Leica CM 3050S, Leica Microsystems, Buffalo Grove, IL) at around −10°C. Micropunches of the left and right amygdala were taken from the appropriate two sections with a 3 mm micropunch. The micropunches were then flash frozen in one tube and stored at −80°C until homogenization. Group sizes consisted of 12 control females, 11 SMG females, 10 control males, and 9 SMG males from 4 different dams and were unrelated to the rats in study 1.

#### Quantification of mRNA

Total RNA was isolated from snap-frozen tissue using an All Prep Mini kit (Cat #80004, Qiagen, Valencia, CA). RNA concentrations were determined using the Qubit Quantification Platform (Cat # Q32857, Invitrogen, Carlsbad, CA) and cDNA was generated with ImProm-II Reverse Transcription System (Cat #A3800, Promega) according to manufacturer recommendations in an Eppendorf MasterCycler personal PCR machine. Samples were stored at −80°C. Real-time RT PCR was conducted with a Stratagene Mx3000P™ real-time PCR system. cDNA was amplified using Sybr ® Green I (S7563, Invitrogen), GoTaq Colorless Master Mix (Cat# M7132, Promega) and ROX as a reference dye (Cat# 12223012, Invitrogen). The amplification protocol is as follows: an initial melting step at 95°C for 2 min followed by 40 cycles of a 95°C melting step for 30 seconds, a 60°C annealing step for 30 seconds, and a 72°C elongation step for 30 seconds. The primers of the gene of interest and the housekeeping gene hypoxanthine phosphoribosyltransferase 1 (HPRT1) (see Table 1) were synthesized by the DNA Synthesis Laboratory, University of Wisconsin Biotechnology Center (Madison, WI). Recent publications indicate that HPRT1 might be one of the best broad application qPCR housekeeping genes, especially when considering genes that would be influenced by hormones [24]–[26]. To date, we have not observed sex differences in HPRT1 expression in any region, treatment, or time point in any of our studies. Primer and sample concentrations were optimized to ensure efficiencies near 100%. Following amplification, a dissociation melt curve analysis was performed to ensure the purity and identity of PCR products. Data were analyzed with the following program term setting based on Invitrogen recommendations: (1) amplified based threshold, (2) adaptive baseline and (3) smoothing moving average with amplification averaging three points. Relative cDNA levels were calculated using the ΔΔC_T_ method [27]. Samples were normalized to HPRT1.

**Table 1:** Primer sequences.

Gene	Forward Sequence 5′ to 3′	Reverse Sequence 5′ to 3′
5HT2a	AACGGTCCATCCACAGAG	AACAGGAAGAACACGATGC
5HT1a	CCGCACGCTTCCGAATCC	TGTCCGTTCAGGCTCTTCTTG
TH	TCTCCCAGGACATTGGACTT	GCACCATAAGCCTTCAGCTC
D1R	CATTCTGAACCTCTGCGTGA	CCATGCTACGCTAATCAGGA
D2R	TCCTGTCCTTCACCATCTCC	CGATGAAGGGCACGTAGAAT
D5R	TGGACCGTTACTGGGCTATT	GGAGATGAGGATGGACAAGG
HPRT1	GCAGACTTTGCTTTCCTTGG	CCGCTGTCTTTTAGGCTTTG

#### Statistical analysis

Statistical comparisons were made between the four treatment groups using a two-way analysis of variance (sex, SMG condition) using SigmaPlot v 11.0 (SPSS Inc., Chicago, Illinois, USA). All post hoc tests were conducted using Tukey HSD.

## Results

### Experiment 1: Variations in maternal touch shape male social play behavior

**Figure 1:**
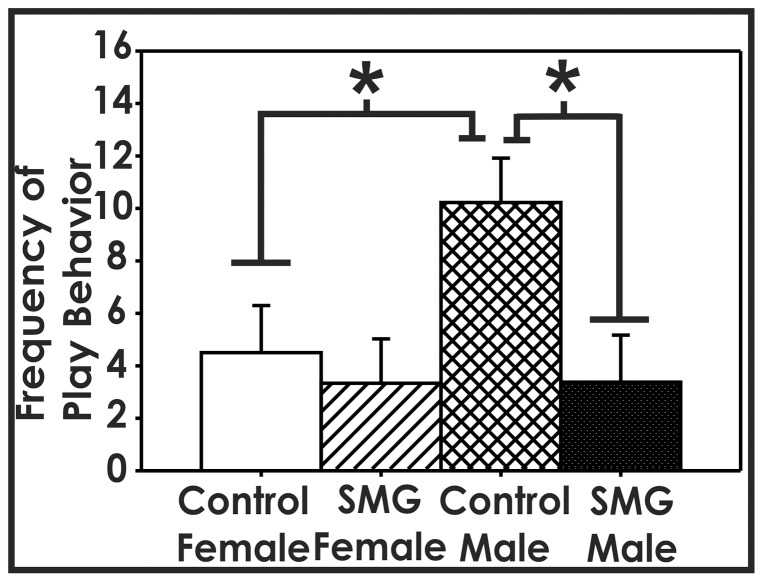
Simulated maternal grooming alters male social play behavior. Between P26 to P29, control-handled males played significantly more than control-handled females (*, p<0.05). Males that were given SMG until P10 engaged in significantly less rough-and-tumble play than control-handled males (*, p<0.01). No difference was found between females who received either control-handling or SMG (p>0.05).

To examine whether additional tactile stimulation alters juvenile social play, we examined the effect of simulated maternal grooming (SMG) on the initiation of play behavior (pinning, pouncing, and biting) between P26 and P29, when juvenile social play begins to peak. Using a two-way ANOVA, we found a significant main effect of group (F(1,33) = 5.275, p<0.05). Post hoc analysis using Tukey HSD revealed that control-handled males played significantly more than control-handled females (p<0.05) and SMG-treated males (p<0.01). There was no difference between the female groups (p>0.05, Figure 1). This suggests that only manipulating somatosensory stimulation associated with maternal contact can shape male juvenile social play behavior.

### Experiment 2: Variations in maternal touch alter neurobiological substrates associated with social play

**Figure 2:**
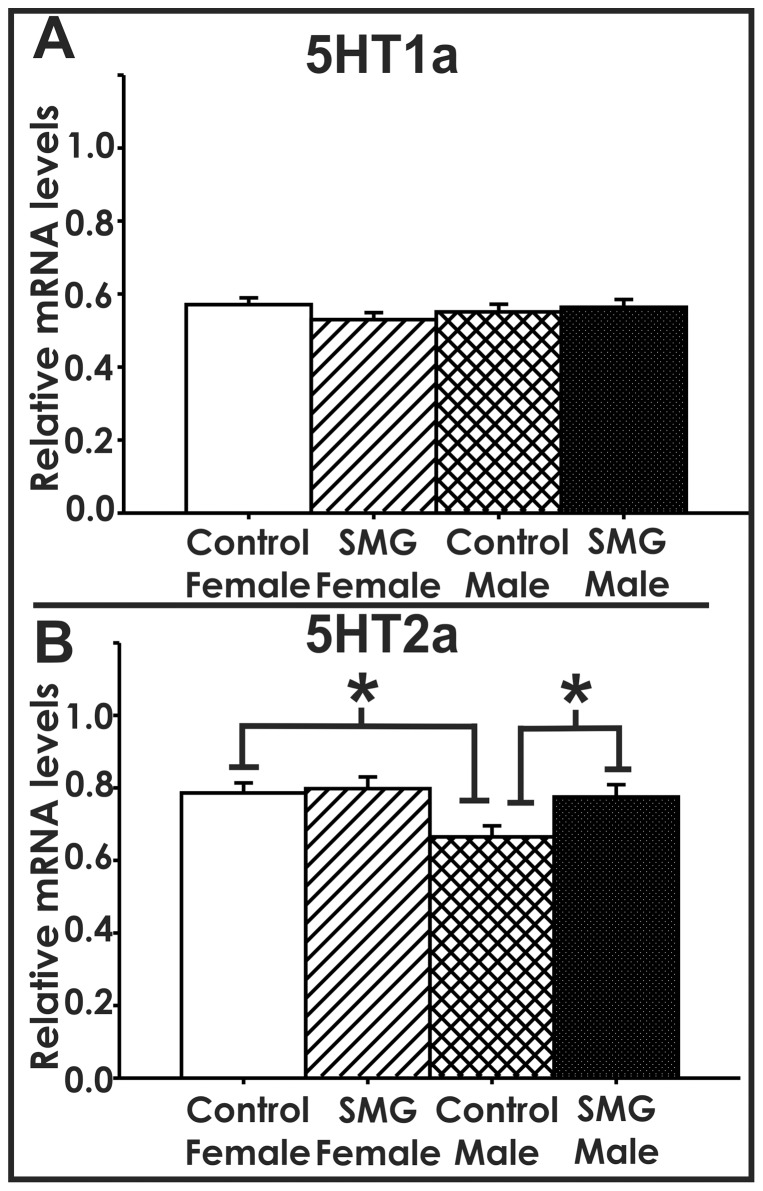
Simulated maternal grooming selectively alters 5HT2a mRNA levels. A) 5HT1a mRNA levels were not altered by SMG or sex (p>0.05). B) There is a sex difference in control-handled animals, where control females have significantly higher levels of 5HT2a than control males (p<0.01). Males that were given SMG until P10 had significantly higher levels of 5HT2a than control-handled males (p<0.05).

To examine if variations in maternal touch influence some of the neurobiological substrates associated with play, we examined the effect of SMG on mRNA levels of serotonin receptors and dopamine system members within the P25 amygdala. When serotonin (or 5-hydroxytryptamine (5HT)) receptor levels were examined, we found that SMG altered serotonin 2a receptor (5HT2a, p<0.05), but not serotonin 1a receptor (5HT1a) mRNA levels (p>0.05, Figure 2). For 5HT2a mRNA levels, there is a significant main effect of sex (F(1,38) = 5.284, p<0.05). Post hoc analysis using Tukey HSD revealed control-handled males have lower 5HT2a mRNA levels than control-handled females (p<0.01) and SMG-treated males (p<0.05, Figure 2B). This suggests that maternal touch selectively upregulates 5HT2a mRNA expression within the juvenile male amygdala.

**Figure 3:**
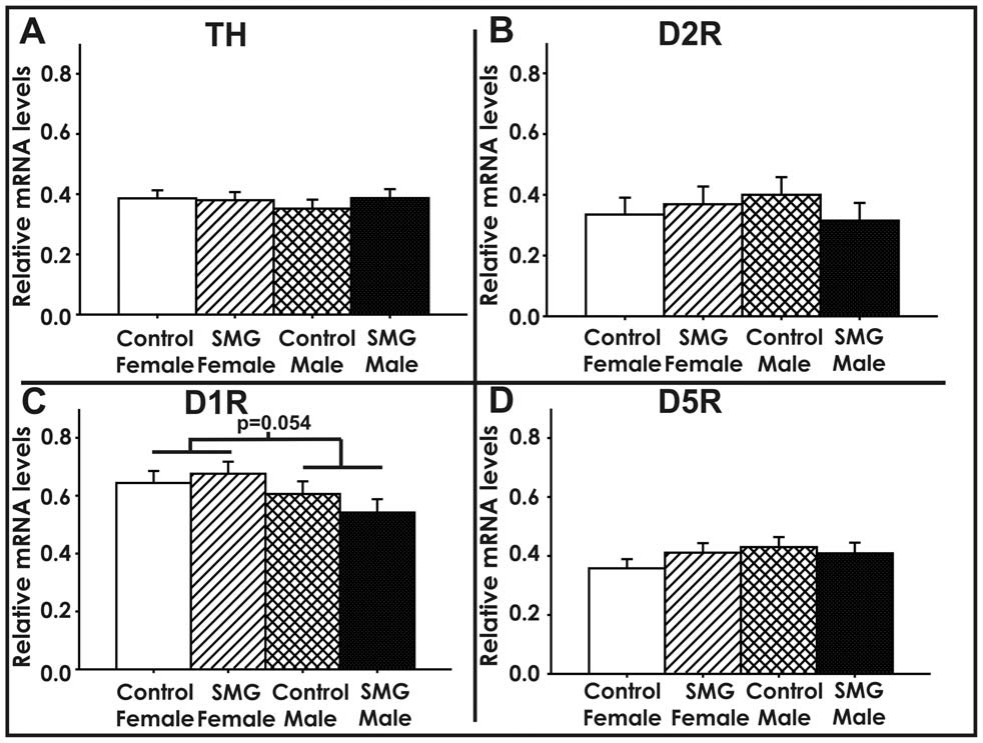
Simulated maternal grooming did not alter mRNA levels of TH, D2R, D1R, D5R. A) TH mRNA levels were not altered by SMG or sex (p>0.05). B) D2R mRNA levels were not altered by SMG or sex (p>0.05). C) D1R mRNA levels were not altered by SMG (p>0.05). An overall trend of females expressing more D1R than males was observed (#, p = 0.054). D) D5R mRNA levels were not altered by SMG (p>0.05).

When members of the dopamine system were examined, we found that SMG did not significantly alter the mRNA levels of tyrosine hydroxylase (TH, p>0.05, Figure 3A), dopamine 2 receptor (D2R, p>0.05, Figure 3B), dopamine 1 receptor (D1R, p>0.05, Figure 3C), or dopamine 5 receptor (D5R, p>0.05, Figure 3D). However, there was a trend for an overall sex difference in D1R mRNA where females tended to express more D1R than males (p = 0.054, Figure 3C). This suggests that maternal touch does not influence the mRNA expression levels of the rate limiting enzyme of dopamine (TH) or dopamine receptors (D2R, D1R, and D5R) within the P25 amygdala

## Discussion

We report that variations in maternal contact during the neonatal period can modify the sex difference typically seen in juvenile social play behavior. By only manipulating the amount of somatosensory stimulation received by the pups, our results lend strong support to the concept that the amounts of infant contact received by the pup can program later juvenile social play behavior. Interestingly, giving extra tactile stimulation to neonatal males decreased juvenile social play compared to control males. Altering the amount of tactile stimuli given to female neonates did not alter their later levels of juvenile social play. These data indicate that differences in the levels of maternal grooming between males and females may act to prevent overt sex differences in juvenile social interactions. Additionally, maternal stimuli selectively altered aspects of the serotonin system within the juvenile male amygdala, but not in females. Specifically, simulating maternal grooming in males eliminated the sex difference observed in 5HT2a mRNA levels. These data suggest that the sex differences in maternal contact during development may act to reduce sex differences in juvenile social play behavior.

The current study artificially upregulated maternal touch via the simulated maternal grooming paradigm. This paradigm allows for other variations in maternal care to be better controlled because we manipulate the pups without treating the dams. This allows each dam to care for a mixed litter of SMG-treated and control-handled pups. Past studies have shown that briefly handling the pups can promote additional licking and grooming when the pups are returned to the litter [28]. However, as the entire mixed litter is removed and returned at the same time, it is unlikely that this would induce a difference in maternal care between treatment groups. Therefore, the current study lends strong evidence to the suggestion that the somatosensory stimulation associated with maternal grooming critically regulates male juvenile social play behavior.

The pattern of juvenile social play in the current study is consistent with past research investigating the influence of maternal care on later juvenile social play behavior. Previous studies indicate that the amount of maternal anogenital licking is promoted by a pup's chemosignals. As such, many of the previous studies that examine the influence of maternal care on social play manipulate the dam's ability to smell in order to reduce the amount of licking and grooming. For example, one study reduced maternal grooming by applying perfume to the pups' anogenital region and found that perfumed males displayed more social play behavior [29]. Similarly, male offspring of dams treated throughout lactation with intranasal zinc sulfate, which reduces chemoperception by the dam and thereby reduced maternal grooming, engaged in more social play than male offspring of control-treated dams [5]. Separating pups from the dam for 3 hours a day for the first two weeks of life also enhanced play-fighting in male juvenile rats [30] [ref]. The development of social play is also influenced by naturally-occurring variations in maternal care. That is, male offspring from dams that exhibited increased levels of pup licking/grooming and arched-back nursing (high LG-ABN mothers) engaged in less social play than males from low LG-ABN mothers [6]. Consistent with our data, none of these studies found that manipulations of maternal care altered the levels of female social play behavior [5], [6], [29]. Although these studies suggest that variations in maternal care influence play behavior, it is important to note that the dams in these studies also displayed other differences in maternal care, like time spent in the nest or the time spent arched-back nursing. Nonetheless, these prior data suggest that that reducing maternal contact increases juvenile social play behavior. Our current data may be consistent with that concept. Specifically, we used the SMG paradigm to mimic an increase in somatosensory stimuli associated with maternal contact. Neonatal males receiving SMG exhibited reduced levels of social play behavior during the juvenile period. While we cannot be control for the possibility that SMG provided to males and females during the neonatal period altered mother-pup interactions, our current data along with the previous findings discussed above suggest that there may be an inverse relationship between maternal licking and grooming and the levels of future juvenile play behavior in males.

Juvenile social play appears to help prepare for adult social behaviors such as male sexual and aggressive behaviors [31]. The actions of a male rat during juvenile social play can be used to predict his aggressiveness as an adult [32]. Preventing males from playing results in abnormal social behaviors, including reduced mating activity [33]. Simulated maternal grooming altered juvenile play behavior only in males. This may suggest that maternal care can help regulate play fighting via an androgen-sensitive pathway. This implication is consistent with research indicating that the amount of anogenital stimulation received by the pup can shape the development of sexual behavior in the male rat. Specifically, reduced maternal licking resulted in males with reduced male sexual behavior [34]. Together, maternal licking reduces juvenile social play but increases male sexual behavior.

As juvenile social play behavior is a complex behavior that is regulated by a multitude of neurochemicals within a variety of brain regions, it is unlikely that maternal care regulates play behavior by modifying only one regulatory factor. It is clear that some of these neurobiological substrates are important for organizing the brain circuitry needed for appropriate social play whereas others are important for appropriately activating the brain at the moment of social play [7], [8]. Altering either the proper organization or activation of these substrates would most likely disrupt normal play behavior. The current study looked for potential disruptions in several neurobiological systems within the amygdala at P25, a time when social play behavior has begun.

Previous data, as well as our current data, suggest an inverse relationship between serotonin signaling and frequency of social play. For example, treatment on the day of testing with a serotonin receptor agonist, quipazine, reduced play behavior [7]. Similarly, acute treatment with compounds that increase central serotonin levels, flouxetine or MDMA, decreased social play behavior [22]. In the current study, we find that the control females, which normally show low levels of juvenile social play, exhibit higher levels of 5HT2a mRNA levels within the amygdala compared to control males. Additionally, SMG-treated males show a reduction in juvenile social play and increased levels of 5HT2a mRNA. Therefore, the pattern of social play and 5HT2a mRNA expression appear inversely related to play behavior. Taken together, these data suggest that the effect of maternal touch on male juvenile social play could be mitigated by altered serotonin signaling in the juvenile amygdala.

As dopamine is involved in motivated behaviors and motor function [35]–[37], several studies have examined whether the dopamine system has a regulatory role for juvenile social play. Indeed, disrupting dopamine synthesis or transmission decreases play behavior [17]–[19]. In the current study, SMG did not alter the mRNA expression levels of the rate limiting enzyme of dopamine (TH) or dopamine receptors (D2R, D1R, and D5R, Figure 3) within the P25 amygdala. Therefore, while maternal contact can program 5HT2a mRNA levels, it does not appear to program dopamine receptor levels within the juvenile amygdala.

The amount of maternal contact each individual pup received while in the home cage was not determined which limits our ability to quantify the difference in anogenital stimulation experienced by SMG and control-handled pups; however this paradigm of simulated maternal grooming has been used for decades to investigate the impact of maternal grooming/contact on offspring development [38]. Simulated maternal contact or grooming is mostly in models of maternal separation and artificial rearing conditions where it can effectively reverse some effects of maternal deprivation. In the current study, we provided SMG or control handling to newborn offspring and returned the litter to a mother in mixed sexes and treatments. While we cannot rule the possibility that the mother could detect SMG vs control handled pups, we did not notice a difference in retrieval rates when a SMG or control handled pup was returned to a cage one at a time in a separate experiment (data not shown). Future studies are needed to determine if dams can distinguish between pups that received simulated maternal grooming and control handling. However, our current data are consistent with previous published data indicating that increased maternal grooming reduces juvenile social play behavior [39], and reduced maternal grooming by application of perfume to neonatal rats increases juvenile social play [40].

In summary, these data indicate that variations in infant contact can lead to lasing changes in juvenile social interactions. Interestingly, providing additional tactile stimuli during early neonatal development appears to lower juvenile rough-and-tumble play behavior in males to female typical levels. Furthermore, additional tactile stimulation during the neonatal period increases the levels of 5HT2a mRNA levels within the amygdala of juvenile males to female typical levels. As serotonin signaling appears to oppose play behavior, these data suggest that sex differences in maternal contact of offspring may serve to reduce overt sex differences in juvenile social interactions. This could suggest that periods of increased care and contact of the offspring may act to reduce sex differences in juvenile behavior; whereas, periods of lower care and contact may exaggerate sex differences in juvenile behavior. These data also indicate that variations in infant contact can have lasting consequences on juvenile social interactions.
